# Sex-Specific Associations Between MTHFR, PPARG and ADRB3 Polymorphisms, Habitual Nutrient Intake and Metabolic Risk in Obesity: A Cross-Sectional Single-Centre Study

**DOI:** 10.3390/nu18152522

**Published:** 2026-08-04

**Authors:** Irina A. Lapik, Inna Yu. Tarmaeva, Dmitry B. Nikityuk

**Affiliations:** 1 Federal Research Centre of Nutrition, Biotechnology and Food Safety, 2/14 Ustinsky Proyezd, Moscow 109240, Russia; 2 I.M. Sechenov First Moscow State Medical University (Sechenov University), Ministry of Health of the Russian Federation, Moscow 119991, Russia

**Keywords:** obesity, nutrigenetics, habitual dietary intake, sex differences, MTHFR C677T, PPARG Pro12Ala, ADRB3 Trp64Arg, arterial hypertension, metabolic dysfunction-associated steatotic liver disease, personalised prevention

## Abstract

**Background/Objectives:** Genetic polymorphisms modulate the risk of metabolic complications of obesity; yet, it remains unclear how genotype reshapes actual dietary intake and why these mechanisms differ between men and women. The aim of this study was to identify sex-specific associations of three common polymorphisms (MTHFR C677T, PPARG Pro12Ala, ADRB3 Trp64Arg) with habitual nutrient intake in patients with obesity, and to explore candidate sex-differentiated dietary hypotheses for future prospective testing. **Methods:** A single-centre cross-sectional study was performed in 348 patients with obesity (83 men, 265 women; BMI ≥ 30 kg/m^2^; age 18–60 years). Genotyping was performed by allele-specific real-time PCR from buccal swabs. Habitual intake of more than 18 nutrients was assessed using a standardised Russian software-based questionnaire. Statistical analysis included Kruskal–Wallis and Mann–Whitney tests with Bonferroni correction (threshold *p* < 0.017), as well as odds ratios (OR) with 95% confidence intervals. Genotype distributions were tested for Hardy–Weinberg equilibrium, and genotype–nutrient associations were additionally examined in models adjusted for age, BMI and total energy intake. **Results:** Three genotype–phenotype associations remained significant after Bonferroni correction. In men, the dominant-heterozygous MTHFR C/T genotype was associated with a hypercaloric dietary pattern—excess protein, fat, sodium and phosphorus—and with a high risk of arterial hypertension (OR 5.96, 95% CI 1.48–24.08; *p* = 0.010). The sodium load in C/T carriers was a consequence of overall overeating rather than a selective preference for salty foods. In men, ADRB3 Trp64Arg carriage was associated with massive dietary cholesterol overconsumption (+466% vs. +153% in Trp/Trp; *p* = 0.012), whereas in women the opposite direction was observed. The PPARG G/G genotype in women was associated with elevated alanine aminotransferase (*p* = 0.027), most pronounced under 40 years (*p* = 0.011), and is considered a marker of hepatic risk without a confirmed dietary target. In women, the MTHFR T/T homozygous genotype showed suggestive associations with excessive mono-/disaccharide intake and a tendency toward hypertension (OR 2.62, 95% CI 1.07–6.43; *p* = 0.035); however, these did not survive FDR correction and require independent validation. **Conclusions:** Genetic polymorphisms were associated with sex-specific patterns of actual dietary intake, a finding that extends current knowledge beyond biochemical risk associations. Based on these associations, we propose testable hypotheses rather than clinical recommendations: that caloric restriction (with consequent sodium reduction) in men with MTHFR C/T, cholesterol restriction in men with ADRB3 Arg64, and mono-/disaccharide restriction in women with MTHFR T/T might reduce metabolic risk; women with PPARG G/G may warrant ALT monitoring. These findings suggest that genotype-informed dietary strategies may need to be sex-specific, and that the window for intervention may be earlier in life than currently practised. However, these hypotheses require prospective interventional validation before any clinical application.

## 1. Introduction

Obesity has reached pandemic proportions worldwide; according to the Global Burden of Disease 2021 forecasts, the number of adults with overweight and obesity is projected to reach 3.8 billion by 2050 [1]. In the Russian Federation the prevalence of obesity among the working-age population exceeds 25% and continues to rise, which determines the high medical and social importance of preventing its complications [2]. The clinical significance of obesity is defined not by excess adiposity per se, but by the spectrum of associated metabolic complications—arterial hypertension, metabolic dysfunction-associated steatotic liver disease (MASLD), dyslipidaemia, hyperuricaemia and disorders of carbohydrate metabolism [3]. Importantly, the risk of these complications differs markedly by sex: in men, cardiovascular and hepatic disturbances predominate, whereas in women oestrogen-dependent metabolic shifts prevail—a pattern we documented in our previous integrative analysis of body composition, biochemical profiles and habitual dietary intake in adults with obesity [4]. Obesity is a disease of multifactorial origin. Alongside dietary factors, genetic predictors make a substantial contribution to the risk of obesity and its complications [5]. The present work is framed within nutrigenetics—the study of how inherited genetic variation shapes individual responses to nutrients and habitual dietary intake—rather than nutrigenomics, which addresses how nutrients modulate gene expression. Among the most extensively studied polymorphisms are rs1801133 of MTHFR (C677T), rs1801282 of PPARG (Pro12Ala) and rs4994 of ADRB3 (Trp64Arg). MTHFR encodes methylenetetrahydrofolate reductase, a key enzyme of folate and homocysteine metabolism; the T-allele reduces enzyme activity and is associated with hyperhomocysteinaemia and endothelial dysfunction [6]. PPARG encodes the nuclear receptor PPARγ, a master regulator of adipogenesis and insulin sensitivity; the minor Ala (G) allele lowers transcriptional activity and, in meta-analysis, is associated with an increased risk of obesity and hypercholesterolaemia [5,7,8]. ADRB3 encodes the β3-adrenergic receptor that mediates lipolysis and thermogenesis in visceral adipose tissue; the Arg64 variant reduces lipolytic activity and has been linked with dyslipidaemia and steatotic liver disease [9]. Accumulating international evidence indicates that the direction and strength of these associations vary between populations and are frequently sex-specific. The same polymorphism may realise risk through different inheritance models in men and women. For MTHFR C677T, the elevated systolic-blood-pressure phenotype in T/T homozygotes is more pronounced in women, with differences of up to 10 mmHg [10]. This attenuation in women is biologically plausible, as oestrogens up-regulate cystathionine-β-synthase activity in the transsulphuration pathway, lowering homocysteine and thereby partly buffering the effect of the T-allele before menopause. In addition, gender-specific MTHFR interactions with serum lipids have been described in a Chinese cohort [11]. For PPARG Pro12Ala, associations with overweight and metabolic phenotypes have only been reported in women in Taiwanese and Brazilian cohorts, but only in men in a Tunisian population [7,12,13]. For ADRB3 Trp64Arg, an association with insulin resistance was observed only in men [9]. Crucially, the effect of these polymorphisms is itself modified by diet: PPARG Pro12Ala interacts with dietary fat intake in relation to body mass index and weight loss [14]. Despite this evidence, most studies examine either genetics or nutrition in isolation. To our knowledge, no study has shown how genotype reshapes actual food consumption—what patients truly eat—differently in men and women, nor which specific nutrient “breakpoints” arise with each genotype and how they might be corrected. This gap is clinically consequential, because the dietary level is the only element of the pathogenic cascade that is directly modifiable by non-pharmacological (nutrigenic) means. Beyond individual candidate variants, precision-nutrition research increasingly integrates genome-wide association findings and polygenic approaches to capture the cumulative genetic contribution to obesity and diet-related traits [5]. The aim of the present study was therefore to evaluate sex-specific associations of the three polymorphisms with habitual dietary intake and to identify sex-specific associations between these common obesity-related polymorphisms, habitual dietary intake and selected metabolic characteristics that may contribute to the development of personalised nutritional strategies in future intervention studies. Recent studies have further demonstrated that common genetic polymorphisms may influence specific components of body composition independently of general adiposity, suggesting that genetic susceptibility may be expressed through alterations in lean tissue, mineral content or regional body composition rather than exclusively through fat accumulation. These findings support the importance of integrating detailed body composition assessment with nutritional and genetic data when investigating obesity-related phenotypes. Consistent with this view, a leptin (LEP) rs7799039 variant was recently associated with total body mineral estimates, but not with fat mass or BMI, in young healthy adults [15], illustrating that genetic variants may shape body composition characteristics even in the absence of detectable differences in adiposity.

## 2. Materials and Methods

### 2.1. Study Design and Participants

A single-centre, cross-sectional (one-time) observational study was conducted at the clinic of the Federal Research Centre of Nutrition, Biotechnology and Food Safety (Moscow, Russia). The study included 348 patients with obesity (83 men, 265 women) aged 18–60 years with a body mass index (BMI) ≥ 30 kg/m^2^. Inclusion criteria were confirmed obesity and signed informed consent. All eligible patients consecutively admitted to the clinic during the study period were invited to participate; recruitment was therefore consecutive rather than by convenience or random selection. Exclusion criteria were severe decompensated comorbidity, pregnancy, and hormonal therapy. All participants were occupationally homogeneous: they belonged to occupational category I according to the Russian physiological labour classification (predominantly intellectual/desk-based work—programmers, accountants, managers), corresponding to a physical activity coefficient of 1.4 × basal metabolic rate, i.e., very low habitual physical activity. No participant reported regular structured exercise at the time of enrolment. This homogeneity, while limiting generalisability to physically active populations, reduces residual confounding by occupational physical activity in the present sex- and genotype-stratified analyses. The recommended nutrient intakes used as the reference for calculating percentage deviations were those specified for this physical activity coefficient (1.4) category. The study was approved by the Ethics Committee of the Federal Research Centre of Nutrition, Biotechnology and Food Safety (Protocol No. 9, 25 December 2023). All participants provided written informed consent before enrolment.

### 2.2. Anthropometry and Body Composition

Height, body weight, BMI, waist and hip circumference and the waist-to-hip ratio were determined. Body composition (fat mass, lean mass, total body water, visceral fat area) was assessed by multi-frequency bioelectrical impedance analysis using the InBody 770 analyser (Biospace, Seoul, Republic of Korea). Body composition was a contextual, not a primary, focus of the present analysis.

### 2.3. Dietary Assessment

For the assessment of habitual dietary intake, the standardised software-based questionnaire “NIAP” (Scientific Nutrition Analysis Tool) was used. The tool was developed by the Federal Research Centre of Nutrition, Biotechnology and Food Safety in collaboration with the company “Nutrient Planner” and is protected by Russian state registration certificate for computer software No. 2023680849; the software is available at https://nplanner.ru. NIAP integrates artificial intelligence algorithms to process 24 h recall and food-frequency components, automatically calculating mean daily intake of more than 18 nutrients: energy, protein, fat, carbohydrates, mono-/disaccharides, dietary fibre, cholesterol, saturated fatty acids (SFA), polyunsaturated fatty acids (omega-3, omega-6, omega-9), sodium, potassium, calcium, magnesium, iron, phosphorus, and vitamins B1, B2, A and C. The AI-driven workflow begins with patient-level observations; algorithms screen a large number of variables and generate results through a process analogous to traditional regression modelling, linking outcomes, covariates and statistical functions. The tool has been extensively tested in clinical practice at the Federal Research Centre of Nutrition, in research institutes and in academic settings. All dietary data refer to habitual home intake prior to hospitalisation. To facilitate comparison across nutrients, intake was expressed as the percentage deviation from the recommended value according to the Russian methodological recommendations on physiological energy and nutrient requirements (MR 2.3.1.0253-21 [16]), applied separately for men and women in line with sex-specific norms; “deficiency” and “excess” were defined as intake below 80% or above 120% of the recommended value, respectively. Where the reference was specified as a range, the midpoint was used. The midpoint of the recommended range was chosen as the most neutral single reference point, consistent with common practice when a point estimate is required for percentage-deviation calculations. The NIAP tool has been adapted for the Russian population, and its outputs are anchored to national reference values (MR 2.3.1.0253-21). However, formal external validation against weighed food records or recovery biomarkers has not yet been published; therefore, all dietary variables should be interpreted as self-reported habitual intake, which is subject to the well-recognised under-reporting of energy intake in individuals with obesity. Sodium was recorded as sodium chloride (g/day) in the clinical dataset and as elemental sodium (mg/day) in the extended dataset; before pooling, all values were converted to elemental sodium (mg/day; 1 g NaCl = 393.4 mg Na) and referred to a single reference of 1300 mg/day. Absolute intakes are reported alongside percentage deviations throughout.

### 2.4. Genotyping

Genotyping of rs1801133 (MTHFR), rs1801282 (PPARG) and rs4994 (ADRB3) was performed by an allele-specific polymerase chain reaction with real-time detection. Buccal epithelium (a non-invasive buccal swab) was used exclusively as the source of genomic DNA for genotyping; no blood sampling was performed for this purpose. Venous blood was drawn separately, and solely for the biochemical measurements described in Section 2.5. All three polymorphisms were in Hardy–Weinberg equilibrium in the sample of 348 genotyped patients (exact test: MTHFR rs1801133 *p* = 0.49; PPARG rs1801282 *p* = 0.17; ADRB3 rs4994 *p* = 0.15). When tested separately by sex, HWE was also confirmed (men: MTHFR *p* = 0.32, PPARG *p* = 0.39, ADRB3 *p* = 0.37; women: MTHFR *p* = 0.53, PPARG *p* = 0.27, ADRB3 *p* = 0.25).

### 2.5. Biochemical Assessment

Venous blood samples were collected after a 12 h fast. Serum biochemical parameters were measured on a KONELAB Prime 60i analyser (Thermo Scientific, Vantaa, Finland): total cholesterol (mmol/L; CV < 2.0%), LDL-cholesterol (mmol/L; CV < 2.5%), HDL-cholesterol (mmol/L; CV < 2.5%), triglycerides (mmol/L; CV < 2.0%), glucose (mmol/L; CV < 2.0%), uric acid (µmol/L; CV < 2.5%), alanine aminotransferase (ALT, U/L; CV < 3.0%), aspartate aminotransferase (AST, U/L; CV < 3.0%), urea (mmol/L; CV < 2.5%), and creatinine (µmol/L; CV < 2.0%). In the extended subset, insulin (µIU/mL; CV < 5.0%), glycated haemoglobin (HbA1c, %; CV < 1.5%), and gamma-glutamyl transferase (GGT, U/L; CV < 3.0%) were additionally measured. Serum vitamins (B2, B6, B12, A, folate, and vitamin D) were determined by standard methods in the extended subset. An ALT above 30 U/L in women and above 40 U/L in men was regarded as a marker of risk for MASLD (metabolic dysfunction-associated steatotic liver disease). Arterial hypertension and coronary heart disease were established from medical records and clinical examination in accordance with current clinical guidelines.

### 2.6. Statistical Analysis

Statistical analysis was performed using Python 3.12 with the pandas and scipy.stats libraries. Normality was tested with the Shapiro–Wilk test; because most variables deviated significantly from normality, data are presented as median and interquartile range (Me [Q1; Q3]). Three genotype groups were compared using the Kruskal–Wallis test, followed by pairwise Mann–Whitney U tests with Bonferroni correction (adjusted significance threshold *p* < 0.017 for three groups). Two independent groups (men vs. women), as well as the dominant (major homozygotes vs. minor-allele carriers) and recessive (minor homozygotes vs. the rest) inheritance models, were compared using the Mann–Whitney U test. To address potential confounding, genotype–nutrient associations were additionally examined using linear regression models adjusted for age, BMI and total energy intake (the latter for nutrient-specific models); genotype–hypertension associations were examined with and without adjustment for age and BMI. Categorical variables were compared using the chi-square test or two-sided Fisher’s exact test. Odds ratios (OR) with 95% confidence intervals were calculated using the ln (OR) ± 1.96 × SE method. Given the small subgroup sizes, all comparisons involving odds ratios were confirmed using two-sided Fisher’s exact test, which is more reliable than asymptotic chi-square tests in small samples. Formal polygenic (allele-sum) scoring was not pursued, because a score weighted on the present sample would be statistically circular and the subgroups were too small for a stable unweighted score; the joint effect of the two loci is therefore reported descriptively only. Statistical significance was set at *p* < 0.05; given the multiple comparisons, associations that survived Bonferroni correction are reported as the most robust, while associations with nominal significance (0.017 ≤ *p* < 0.05) are considered hypothesis-generating and require independent confirmation. Given the large number of comparisons across sexes, age strata, genotypes and nutrients, the Benjamini–Hochberg false-discovery-rate (FDR) procedure was applied separately within each pre-specified family of comparisons, reflecting the hierarchical structure of the analyses: MTHFR–nutrient associations in men; MTHFR–nutrient associations in women; ADRB3–nutrient associations and biochemical-marker associations (ALT, uric acid, vitamin B6, GGT). Associations with FDR q < 0.05 were considered robust; those with q ≥ 0.05 are reported as exploratory and hypothesis-generating. However, given the exploratory nature of this study, we prioritised reporting all nominally significant associations with appropriate caveats, and all such associations that did not survive Bonferroni correction are regarded as hypothesis-generating. To address the overall multiplicity of testing, a small set of pre-specified primary (confirmatory) endpoints was distinguished from secondary (exploratory) analyses. The primary endpoints were the two genotype–nutrient associations that survived Bonferroni correction (MTHFR C/T with energy/sodium intake in men, and ADRB3 Trp64Arg with dietary cholesterol in men). All remaining comparisons—across three polymorphisms, two sexes, more than 18 nutrients, three inheritance models, biochemical variables, clinical outcomes and age strata (well over 300 individual tests)—are secondary and hypothesis-generating and require independent replication. Because several genotype subgroups were small after sex stratification, a post hoc power assessment showed that only large effects were detectable: at α = 0.05 and 80% power the minimum detectable odds ratios were approximately 20 for male T/T, 10 for male Arg64 and 4.6 for female PPARG G/G carriers. Accordingly, null findings in these subgroups must not be interpreted as evidence of no effect. For continuous outcomes (nutrient intakes), post hoc power calculations indicated that in the smallest subgroups (male T/T, *n* = 9; male Arg64, *n* = 12; female G/G, *n* = 18) the minimum detectable effect size (80% power, α = 0.05) corresponded to approximately 0.7–1.0 pooled standard deviations; thus only very large differences could be detected in these groups, and smaller but potentially meaningful effects may have been missed. The study is reported in accordance with the STROBE guidelines for observational studies.

## 3. Results

### 3.1. General Characteristics of the Study Population

The general characteristics of the clinically documented cohort are presented in Table 1. Men and women were comparable in age (median 37 years in both groups). Statistically significant sex differences were observed for anthropometric and biochemical parameters: men had higher BMI, uric acid, ALT, AST, triglycerides and basal metabolic rate, whereas HDL-cholesterol and the fat-mass fraction were higher in women. The distribution of the studied genotypes did not differ between the sexes (*p* > 0.37 for all three polymorphisms). Genotype frequencies in the full analytic sample (*n* = 348) were: MTHFR rs1801133—C/C 48.0%, C/T 41.4%, T/T 10.6%; PPARG rs1801282—C/C 63.2%, C/G 30.2%, G/G 6.6%. ADRB3 rs4994—Trp/Trp 77.6%, Trp64Arg 22.4%.

### 3.2. Sex-Specific Baseline Dietary Patterns

**Figure 1:**
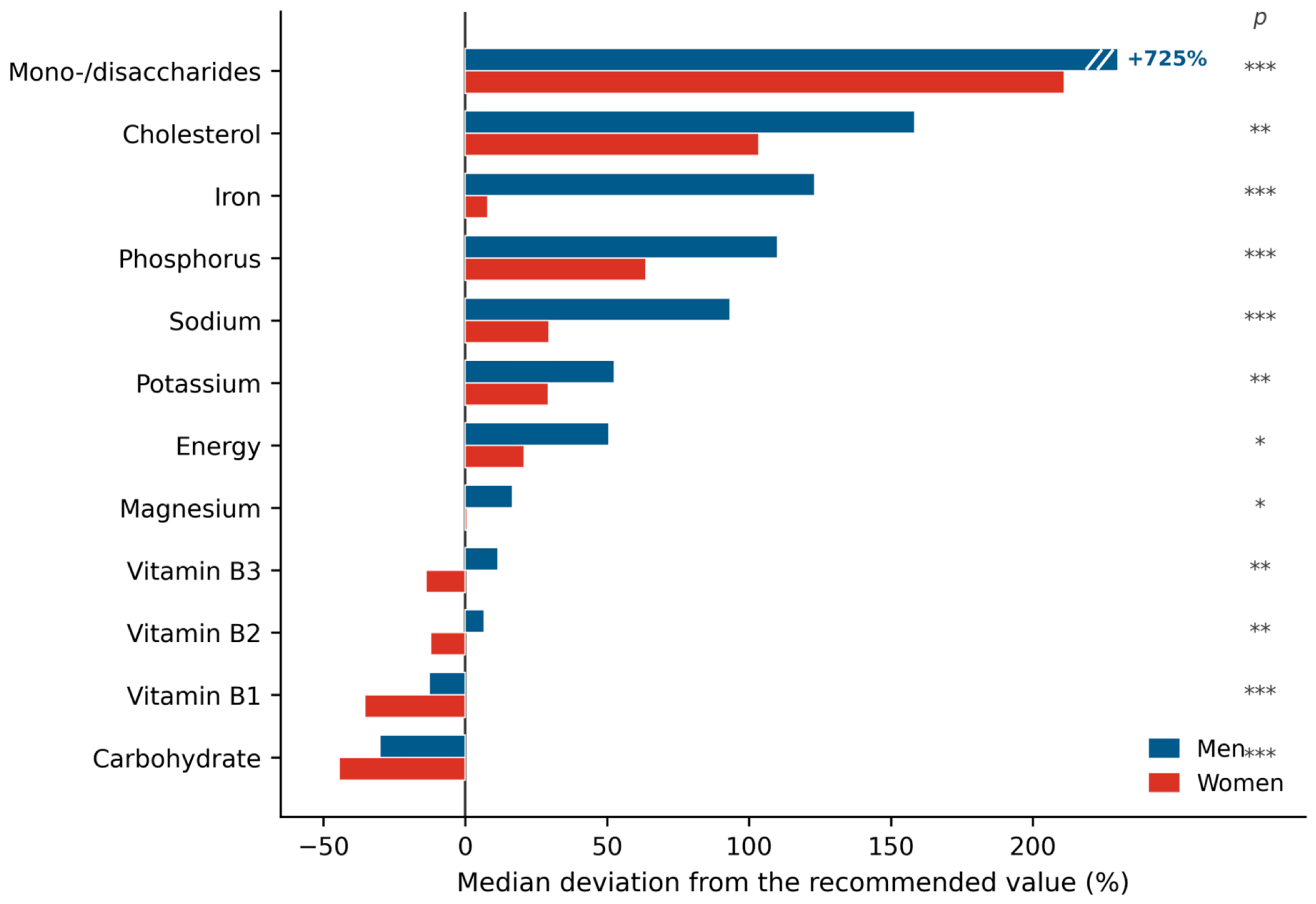
Sex differences in habitual nutrient intake in patients with obesity. Bars show the median deviation from the recommended value (%) for the twelve nutrients that differed significantly between men and women (all *p* < 0.05, Mann–Whitney U test). Bars exceeding +260% are truncated and the value is printed. * *p* < 0.05; ** *p* < 0.01; *** *p* < 0.001.

Independent of genotype, comparison of habitual intake between men and women with obesity revealed statistically significant differences. Men showed a significantly greater excess over recommended intake for iron, sodium, phosphorus, potassium, saturated fatty acids, dietary cholesterol, magnesium and vitamin B2 (*p* < 0.05 for all; Figure 1). On recomputation, saturated fatty acids no longer differed significantly (*p* = 0.098), whereas energy (*p* = 0.014), carbohydrate (*p* < 0.001), mono-/disaccharides (*p* < 0.001) and vitamins B1 and B3 (both *p* < 0.002) did differ. No significant sex difference was found for protein, fat, dietary fibre, calcium, omega-3 and omega-6 fatty acids or vitamins A and C. This baseline sex asymmetry provides the context on which the genotype-specific effects described below are superimposed.

### 3.3. MTHFR rs1801133: Sex-Specific Dietary Phenotypes

#### 3.3.1. Men

**Figure 2:**
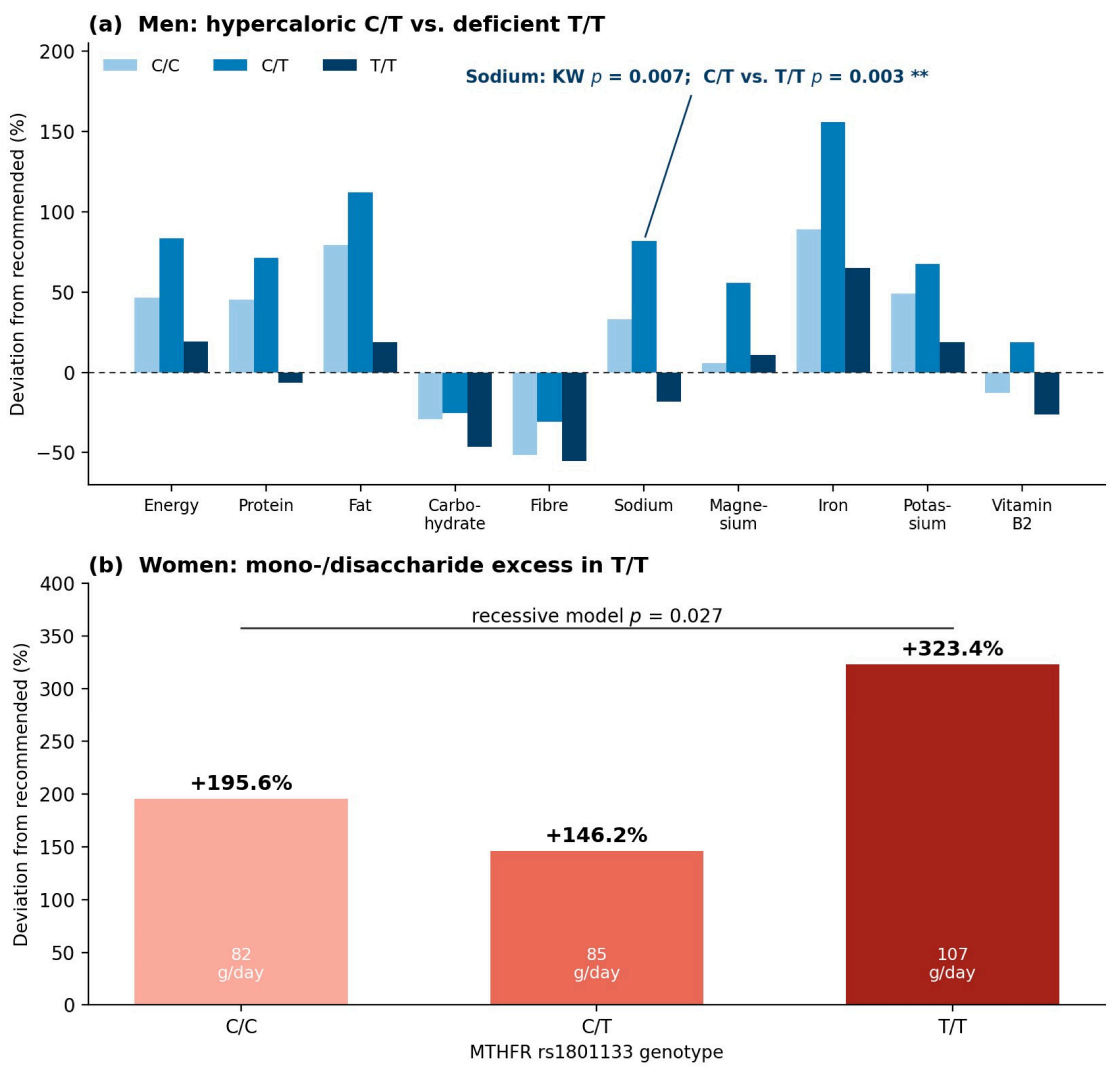
MTHFR rs1801133 (C677T) sex-specific dietary phenotypes. (**a**) In men, C/T carriers show a hypercaloric protein–fat pattern, whereas T/T carriers show a macro-/micronutrient deficit. (**b**) In women, T/T carriers show a marked excess of mono-/disaccharides (+323.4% vs. +195.6% in C/C; recessive model *p* = 0.027). The dashed line marks the recommended value (0% deviation). ** *p* < 0.017 (Bonferroni-corrected for the pairwise comparison C/T vs. T/T in men).

In men (*n* = 83), MTHFR genotype was associated with intake of several nutrients (Table 2 and Table 3). Heterozygous C/T carriers displayed a hypercaloric pattern with excess protein (+71.1%; 148 g/day), fat (+111.9%; 186 g/day) and sodium (+134.3%; 3047 mg Na/day, equivalent to 7.7 g of salt). By contrast, T/T homozygotes showed a generalised deficient pattern: reduced protein (−6.6%), lower excess of fat (+18.6%) and magnesium (+17.2%), and reduced vitamin B2 (+26.4%). The strongest signal was sodium: Kruskal–Wallis *p* = 0.007, with the pairwise contrast C/T vs. T/T (3047 vs. 1609 mg Na/day) reaching *p* = 0.003 and surviving Bonferroni correction (threshold *p* < 0.017). Under the recessive model only protein reached nominal significance (*p* = 0.043). Dietary phosphorus followed the same C/T-linked pattern (C/T +127.7% vs. T/T +39.7%; Kruskal–Wallis *p* = 0.033, with the pairwise contrast C/T vs. T/T reaching *p* = 0.012 and surviving Bonferroni correction), consistent with the higher intake of protein-rich, phosphorus-dense foods in C/T carriers. Magnesium, iron and potassium, previously reported as significant, had inadvertently been computed on the 37 men of the clinical cohort rather than on all 83; recomputed on the full sample they lose significance (*p* = 0.68, 0.21 and 0.13, respectively). Nevertheless, the consistent directionality across multiple macro- and micronutrients supports the biological coherence of the observed hypercaloric pattern in C/T carriers. The T/T male subgroup is small (*n* = 9), so all contrasts involving it are exploratory and the corresponding estimates are imprecise. Thus, in men the T-allele is associated with two opposite nutrient phenotypes: a hypercaloric protein–fat pattern in C/T and a macro-/micronutrient deficit in T/T (Figure 2a).

#### 3.3.2. Women

In women (*n* = 265) the pattern differed qualitatively from men. The most pronounced finding was a marked excess of mono-/disaccharides in T/T homozygotes: 107 g/day (median deviation +323.4%) vs. 82 g/day (+195.6%) in C/C and 85 g/day (+146.2%) in C/T (recessive model T/T vs. C/C + C/T, *p* = 0.027 (Benjamini–Hochberg FDR q = 0.189 across the 12 tests in this family); Table 4 and Table 5, Figure 2b). This does not cross the Bonferroni threshold of *p* < 0.017 and is therefore exploratory. The dietary-fibre deficit was less severe in C/T and T/T carriers than in C/C (dominant).

### 3.4. MTHFR rs1801133: A Male-Specific Sodium Excess

**Figure 3:**
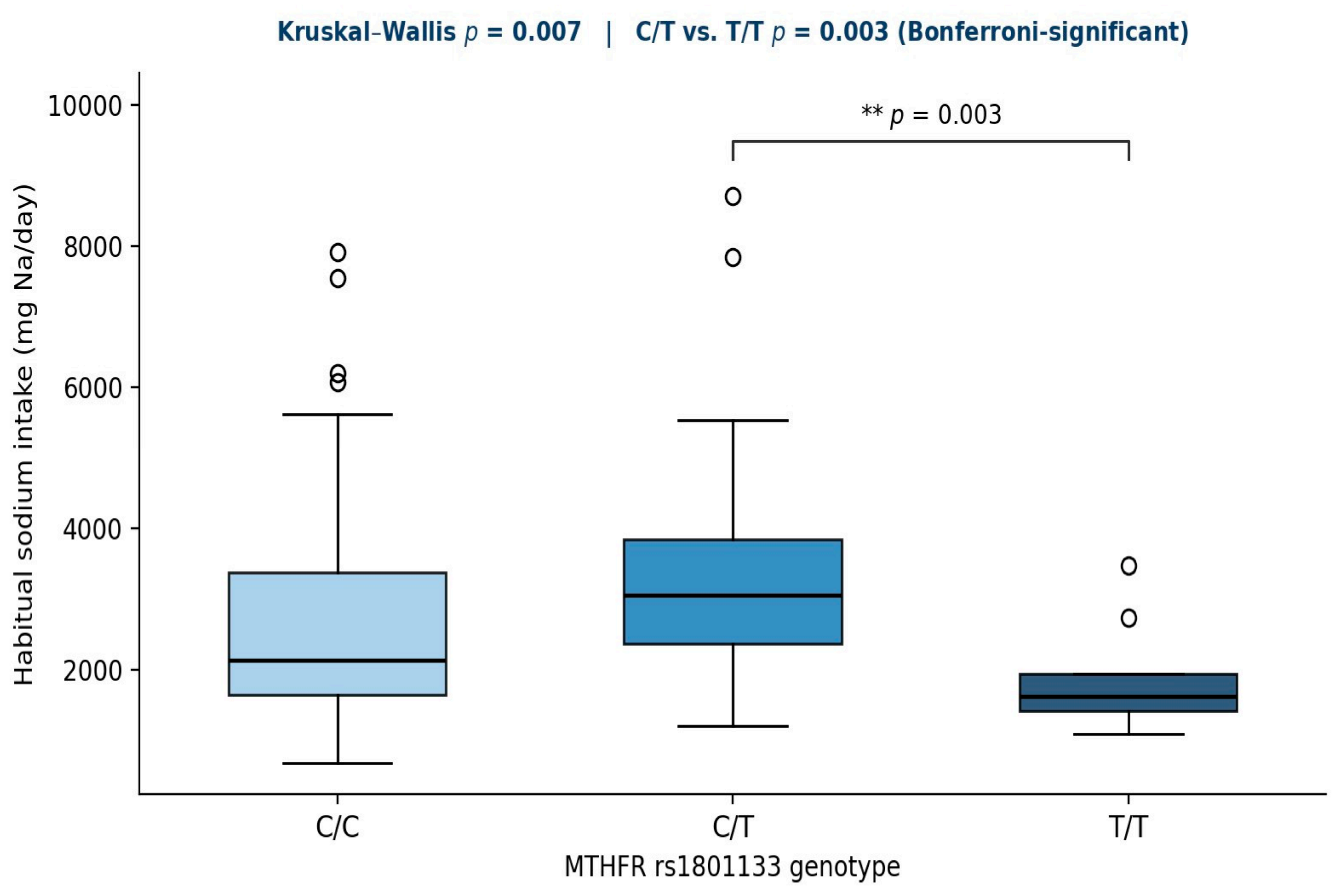
MTHFR rs1801133 and habitual sodium intake in men with obesity (elemental sodium, mg/day; reference 1300 mg/day). Heterozygous C/T carriers show a marked sodium excess (median 3047 mg/day) relative to C/C (2128 mg/day) and T/T (1609 mg/day). Kruskal–Wallis *p* = 0.007; C/T vs. T/T *p* = 0.003, surviving Bonferroni correction. Boxes show median and interquartile range; whiskers show 1.5 × IQR; dots show individual participants; and dashed line shows the recommended intake.

Because sodium was recorded in different units in the two datasets (sodium chloride, g/day, in the clinical cohort; elemental sodium, mg/day, in the extended cohort), all values were first converted to a common scale (elemental sodium, mg/day; 1 g NaCl = 393.4 mg Na) and referred to a single reference of 1300 mg/day. After this harmonisation, absolute habitual sodium intake in men was associated with MTHFR rs1801133 genotype (Kruskal–Wallis *p* = 0.007): heterozygous C/T carriers showed a marked sodium excess (median 3047 mg Na/day, +134% above the reference) compared with C/C (2128 mg/day) and T/T homozygotes (1609 mg/day). The pairwise contrast C/T vs. T/T reached *p* = 0.003 and survived Bonferroni correction (threshold *p* < 0.017; Figure 3), and the dominant-heterozygous contrast C/T vs. C/C + T/T was likewise significant (*p* = 0.007).

Because absolute sodium intake was strongly correlated with total energy intake (Spearman ρ = 0.61, *p* < 0.001), we tested whether the sodium excess reflected a saltier diet or simply a larger one. Sodium density (mg Na per 1000 kcal) did not differ across MTHFR genotypes in men (721, 800 and 637 mg/1000 kcal for C/C, C/T and T/T; Kruskal–Wallis *p* = 0.22), and in a linear model adjusting for energy intake the C/T effect on absolute sodium was abolished (β = +334 mg, *p* = 0.39), whereas energy remained a strong predictor (*p* < 0.001). The C/T-linked sodium excess is therefore part of a generalised hypercaloric pattern rather than a selectively saltier diet—C/T men eat more food overall, not more salt per calorie. This does not diminish its clinical relevance: it is the absolute sodium load (3047 vs. 1609 mg/day) that drives blood pressure, and it co-segregates with the same genotype that carries the male hypertension risk, providing a dietary link between MTHFR C/T and blood pressure that operates through overall overconsumption. In a logistic regression model additionally adjusted for age and BMI, the MTHFR C/T–hypertension association in men remained statistically significant and consistent with the unadjusted estimate (OR 5.96, 95% CI 1.48–24.08; *p* = 0.010), indicating that the C/T genotype contributes to hypertension risk independently of these demographic covariates. A sodium association with PPARG rs1801282 in women, reported in a preliminary analysis, was confined to the 77 women of the extended cohort (dominant model *p* = 0.025, only eight G/G carriers) and was absent in the clinical cohort (*p* = 0.86); after unit harmonisation it did not persist (Kruskal–Wallis *p* = 0.56) and is not carried forward. No other macro- or micronutrient was significantly associated with PPARG rs1801282 in either sex. To systematically confirm the absence of dietary associations, we compared habitual nutrient intakes across PPARG genotypes separately in women (Table 6) and men (Table 7). No significant differences were observed for any nutrient in either sex (all *p* > 0.05, Kruskal–Wallis test), including energy, protein, fat, carbohydrates, mono-/disaccharides, cholesterol, sodium and other micronutrients. These negative findings are important because they rule out the possibility that the elevated ALT in female PPARG G/G carriers (Section 3.7) is driven by differences in dietary intake. Thus, PPARG G/G is retained solely as a biochemical marker of hepatic risk rather than as a determinant of dietary behaviour.

**Figure 4:**
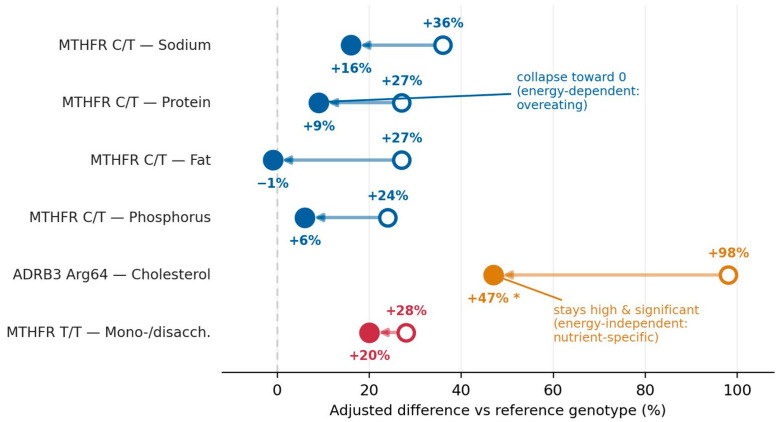
Effect of adjusting for total energy intake on the principal genotype–nutrient associations. For each association the crude (open circle) and energy-adjusted (filled circle) percentage difference versus the reference genotype is shown, with an arrow indicating the shift on adjustment. The MTHFR C/T macronutrient and sodium excesses collapse toward zero after energy adjustment (energy-dependent, reflecting overall overeating), whereas the ADRB3 Trp64Arg cholesterol excess remains large and statistically significant (energy-independent, nutrient-specific); the female MTHFR T/T mono-/disaccharide excess is largely retained. * *p* < 0.05.

To formally test whether these genotype–nutrient associations were independent of total energy intake, log-transformed nutrient intakes were regressed on genotype without adjustment, with adjustment for age and BMI, and with adjustment for log total energy intake (Table 8); coefficients are the adjusted percentage difference for the risk genotype versus the reference group. In men, all four MTHFR C/T excesses (sodium +36%, protein +27%, fat +27%, phosphorus +24%; all crude *p* < 0.05, confirmed by rank tests in Table 2, Table 3, Table 4, Table 5, Table 6, Table 7 and Table 8) were essentially unchanged after adjustment for age and BMI (e.g., fat +31%) but were abolished after adjustment for total energy intake (fat +31% → −1%), confirming that the C/T ‘hypercaloric’ pattern reflects overall overeating rather than nutrient-specific preferences. By contrast, the ADRB3 Trp64Arg cholesterol excess in men remained significant through all models, including full energy adjustment (+47%, 95% CI +3 to +109; *p* = 0.035), indicating a genuinely energy-independent, nutrient-specific effect. In women, the MTHFR T/T mono-/disaccharide excess was significant (Mann–Whitney *p* = 0.032) and, in contrast to the male macronutrient pattern, retained its magnitude after energy adjustment (+20%), consistent with a more nutrient-specific ‘sugar’ phenotype (Figure 4).

### 3.5. ADRB3 rs4994: The “Mirror” Effect on Dietary Intake

**Figure 5:**
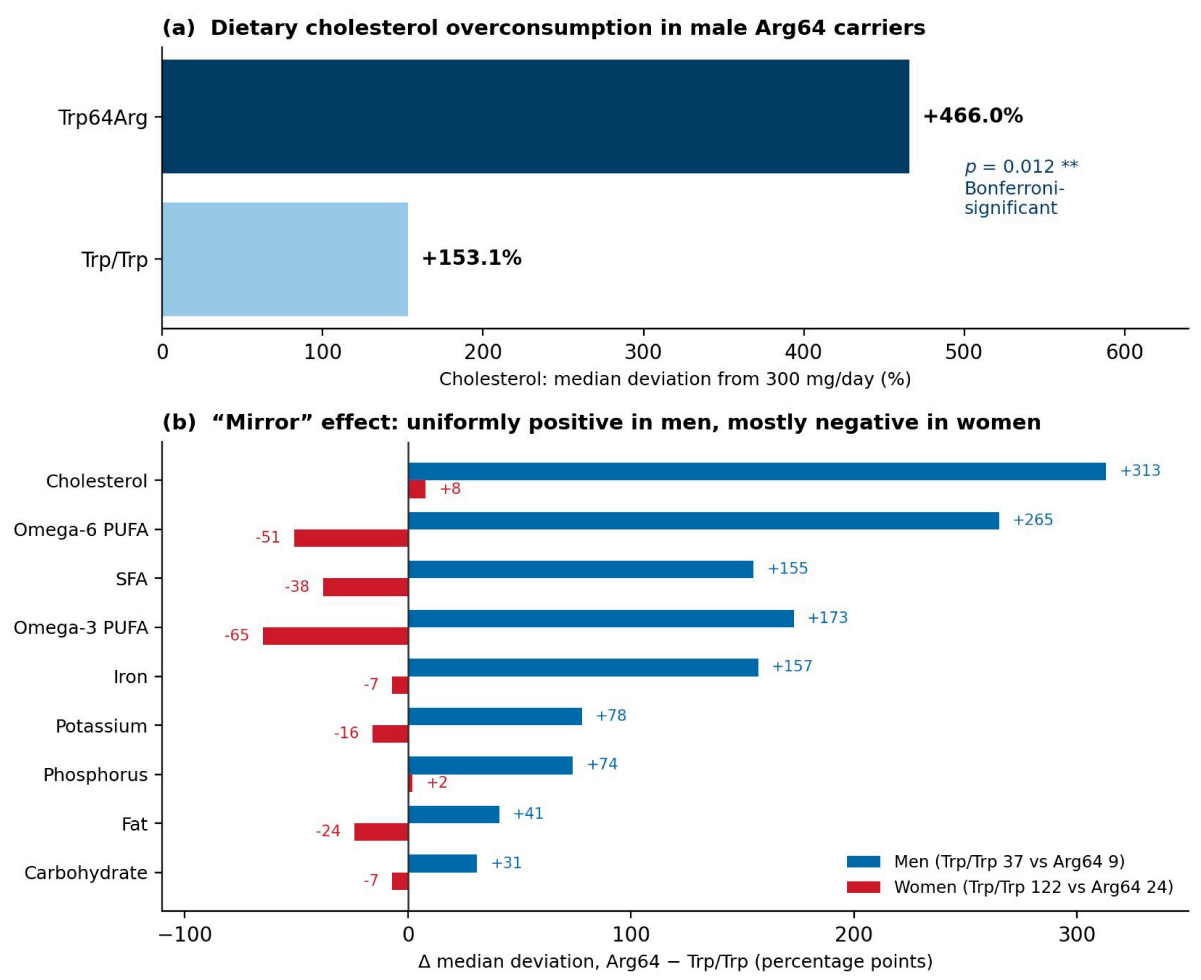
ADRB3 rs4994 (Trp64Arg) sex-specific “mirror” effect on dietary intake. (**a**) In men, dietary cholesterol reaches +466% in Trp64Arg vs. +153% in Trp/Trp (*p* = 0.012, Bonferroni-significant). (**b**) Diverging Δ (Arg64 − Trp/Trp) across nutrients: uniformly positive in men, uniformly negative in women. ** *p* < 0.017 (Bonferroni-corrected for pairwise comparisons).

ADRB3 rs4994 produced a strikingly sex-divergent dietary pattern (Table 9 and Table 10, Figure 5). In men, intake was higher in Trp64Arg carriers than in Trp/Trp for all 21 nutrients examined; in women the same allele was associated with lower intake for 16 of 21 nutrients. The strongest and only Bonferroni-significant association in men was dietary cholesterol: 849 mg/day [429; 1005] in Trp64Arg (*n* = 9) vs. 380 mg/day [201; 607] in Trp/Trp (*n* = 37), against a recommended intake of 300 mg/day; this corresponds to a median deviation of +466.0% vs. +153.1% (Δ = +313 percentage points; *p* = 0.012). In women the corresponding intakes were 339 vs. 327 mg/day (*p* = 0.89). Trends in men (*p* < 0.10) were also seen for iron (Δ +157%; *p* = 0.055) and carbohydrate (Δ +31%; *p* = 0.055); a weaker trend was present for omega-6 PUFA (Δ +265%; *p* = 0.091). In women, no single nutrient reached significance, but the consistently inverse direction across 16 of 21 nutrients defines a sex-specific “mirror” modification of the nutrient pattern in Arg64 carriers: hyperphagic in men, hypophagic in women. After FDR correction across all nutrient comparisons in women, none of the ADRB3 associations reached q < 0.05 (all q > 0.15), indicating that the consistent directional pattern does not constitute statistically robust evidence for nutrient-specific effects in women.

### 3.6. Sex-Specific Inheritance of Hypertension Risk

**Figure 6:**
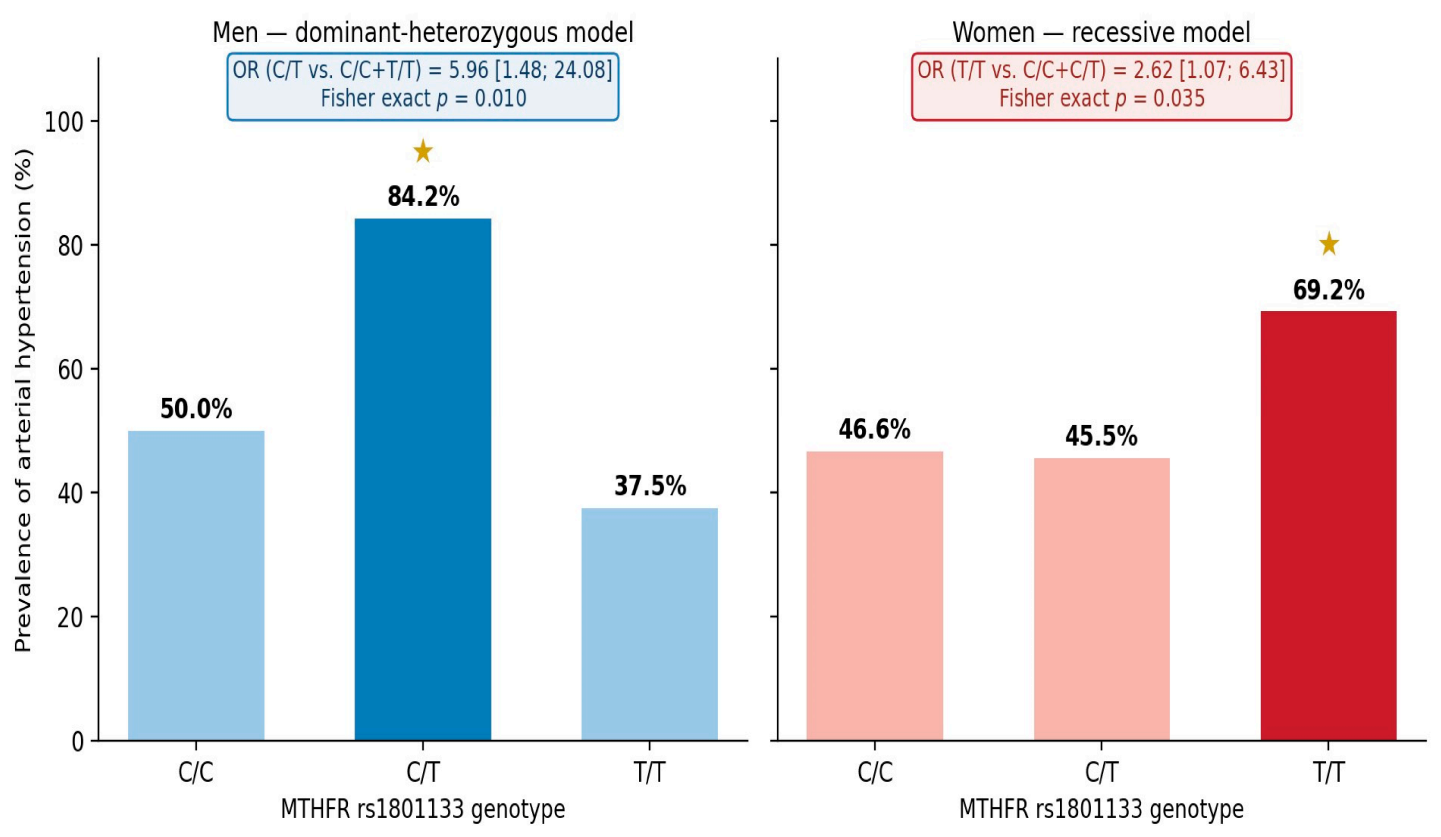
Sex-specific inheritance of hypertension risk for MTHFR rs1801133. In men the risk genotype is the heterozygote C/T (dominant-heterozygous model); in women it is the homozygote T/T (recessive model). Error bars are 95% Wilson confidence intervals; stars mark the significant risk genotype in each sex.

MTHFR rs1801133 was associated with hypertension through different risk genotypes in men and women. In men, C/T heterozygotes had hypertension in 84.2% of cases (16/19), more than C/C (48.3%, 14/29) or T/T (37.5%, 3/8) carriers (χ^2^ *p* = 0.024); the OR for C/T vs. pooled homozygotes was 5.96 [1.48; 24.08] (Fisher exact *p* = 0.010)—a dominant-heterozygous model. In a sensitivity analysis excluding the small male T/T subgroup (*n* = 9), the C/T–hypertension association remained significant (C/T vs. C/C only: OR 5.33, 95% CI 1.27–22.48; Fisher exact *p* = 0.029), indicating that the finding is not driven by the small homozygous group. In women the maximum risk was linked to T/T homozygosity: hypertension frequency was 69.2% in T/T, 45.5% in C/T and 46.6% in C/C (χ^2^ *p* = 0.095); the OR for T/T vs. pooled C-allele carriers was 2.62 [1.07; 6.43] (Fisher exact *p* = 0.035)—a recessive model (Figure 6). In a logistic regression model adjusted for age and BMI, the direction of the female T/T–hypertension association was preserved and consistent with the unadjusted estimate (OR 2.62, 95% CI 1.07–6.43; *p* = 0.035). Because this association did not survive Bonferroni correction and rests on a small T/T subgroup, it should be regarded as suggestive and requires independent validation. After FDR correction across the hypertension comparisons in women this association yielded q = 0.078, further emphasising its exploratory nature. Age stratification strengthened these patterns: in men below 40 years the C/T–hypertension OR reached 9.00 [1.60; 50.69] (*p* = 0.011), and in women below 40 years the T/T OR reached 5.52 [1.42; 21.45] (*p* = 0.014). Above 40 years the genotype differences were attenuated by age-related “saturation” of risk.

Notably, the 95% confidence intervals for these ORs were wide (e.g., 1.48–24.08 for the overall male C/T effect and 1.60–50.69 for the under-40 subgroup), reflecting the limited sample size of genotype-stratified and age-stratified subgroups. These estimates are therefore exploratory and require validation in larger independent cohorts before any quantitative interpretation can be made. In a logistic regression model adjusted for age and BMI, the male C/T–hypertension association remained statistically significant (adjusted OR 6.14, 95% CI 1.48–25.53; *p* = 0.013; event counts 16/19 in C/T vs. 17/36 in pooled homozygotes). On additional adjustment for total energy intake the point estimate attenuated (adjusted OR 3.31, 95% CI 0.69–15.86; *p* = 0.13), suggesting that total energy intake may act as a mediator on the causal pathway rather than as a confounder; this attenuation is therefore expected and does not negate the association (multivariable models summarised in Table 11).

### 3.7. PPARG rs1801282, ADRB3 rs4994 and Liver/Purine Markers

In women, PPARG G/G (Ala/Ala) was associated with elevated ALT (median 31.0 U/L vs. 17.0 U/L in C/C; Kruskal–Wallis *p* = 0.027, FDR q = 0.054 across the biochemical-marker family; exploratory), most pronounced below 40 years (31.5 vs. 16.0 U/L; *p* = 0.011), identifying G/G as a marker of hepatic risk in young women—consistent with the sodium-excess dietary phenotype and with RAAS/hepatic mechanisms. ADRB3 rs4994 showed a biochemical “mirror” paralleling the dietary one: in men, Trp64Arg carriers tended to have higher ALT (47.5 vs. 35.0 U/L; *p* = 0.058), while in women they had significantly lower uric acid (302.0 vs. 344.5 µmol/L; *p* = 0.040). In the extended panel, male T-allele carriers of MTHFR had lower serum vitamin B6 (8.04 vs. 11.26 µg/L; *p* = 0.004, FDR q = 0.016; robust as an exploratory finding)—a biochemical correlate of the homocysteine hypothesis, vitamin B6 being a cofactor of cystathionine-β-synthase in the trans-sulphuration pathway.

### 3.8. ADRB3 Adds No Independent Hypertension Signal in Men

In men, ADRB3 Trp64Arg did not carry an independent hypertension signal (58.3% in Arg64 carriers vs. 60.5% in Trp/Trp; Fisher exact *p* = 1.0). Within MTHFR C/T carriers, the addition of ADRB3 Arg64 did not further increase hypertension risk (86.7% in C/T + Arg64 vs. 75.0% in C/T without Arg64), although the small number of C/T carriers without Arg64 (*n* = 4) precludes firm conclusions. Thus, the cardiovascular risk signal in men is attributable to MTHFR C/T alone, while ADRB3 Arg64 marks a distinct dietary target (cholesterol) without an additive effect on hypertension. The weighted polygenic score has been removed to avoid circularity, and no polygenic model is carried forward.

### 3.9. Sex × Genotype Map of Complications and the “Prevention Window” Below 40 Years

**Figure 7:**
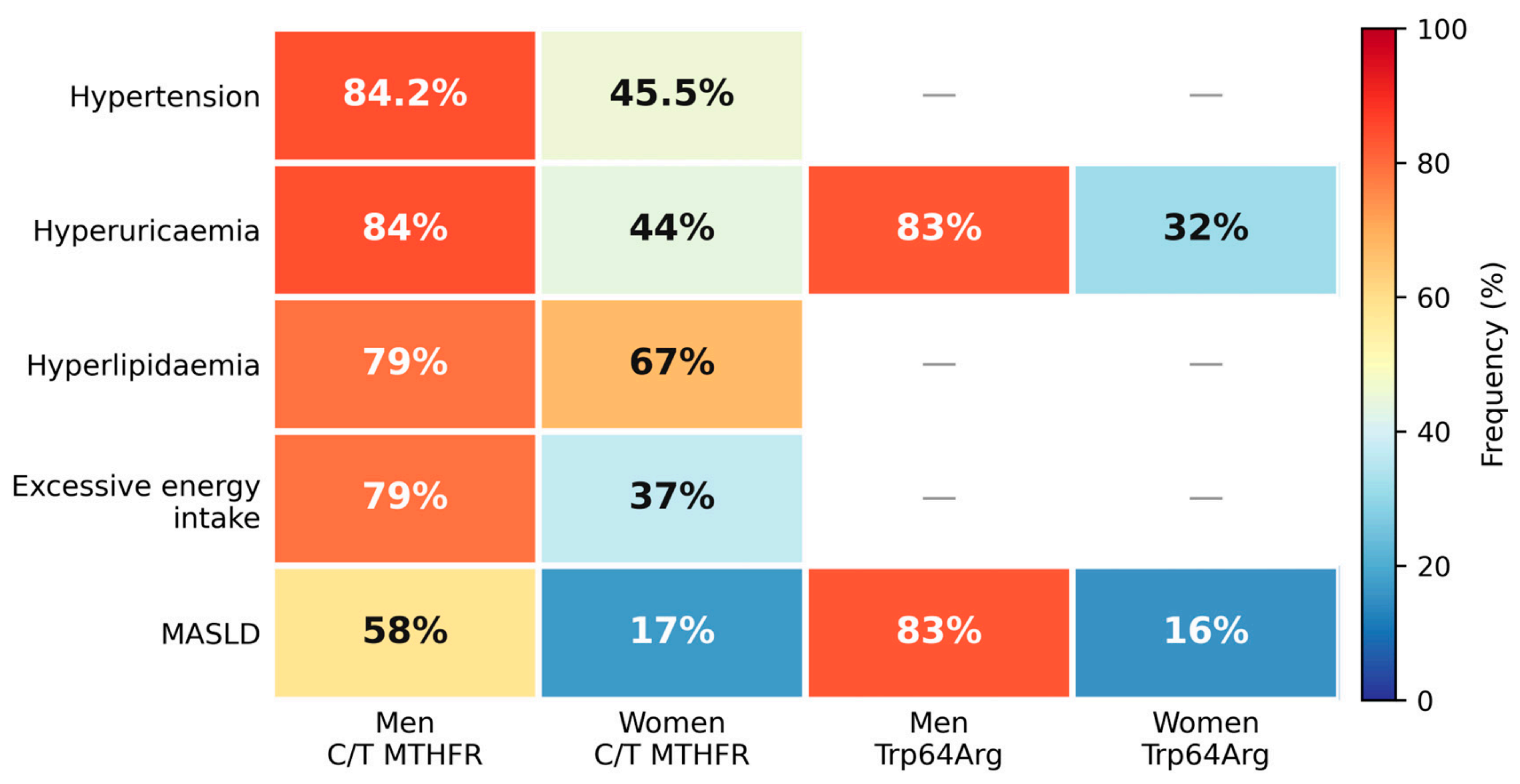
Sex × genotype map of associated metabolic phenotypes and clinical outcomes in patients with obesity: frequency (%) of each phenotype by risk genotype and sex. Colour intensity is proportional to frequency. MASLD, metabolic dysfunction-associated steatotic liver disease.

**Figure 8:**
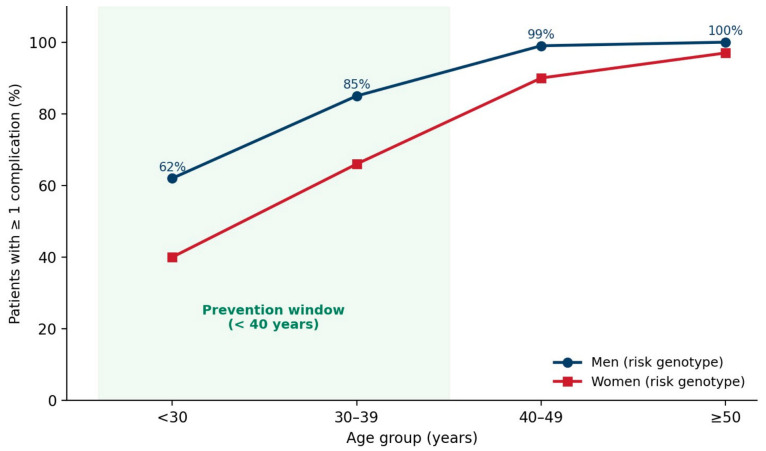
Age dynamics of metabolic complications by sex in risk-genotype carriers. The shaded band (<40 years) marks the priority prevention window.

A sex × genotype map of realised complications (Figure 7) demonstrated pronounced sex asymmetry: for the same risk genotype, men realised complications far more frequently than women. Among C/T MTHFR carriers, hypertension, hyperuricaemia, elevated LDL-C, and excessive energy intake occurred in 79–84% of men vs. 37–67% of women; among Trp64Arg ADRB3 carriers, elevated ALT and hyperuricaemia reached 83% in men vs. 16–32% in women. Age stratification (Figure 8) showed that in men carrying any risk genotype the frequency of ≥1 complication exceeded 95–100% already after 30 years, whereas in women it was substantially lower below 30 years and rose sharply to 79–100% by 30–39 years. These data define the age below 40 years—especially for female T/T MTHFR carriers—as the priority “window” in which nutrigenic correction can prevent or delay complications, and support molecular-genetic screening at 25–40 years in newly diagnosed obesity.

### 3.10. A Hypothesised Gene–Diet–Disease Cascade (Conceptual Model)

**Figure 9:**
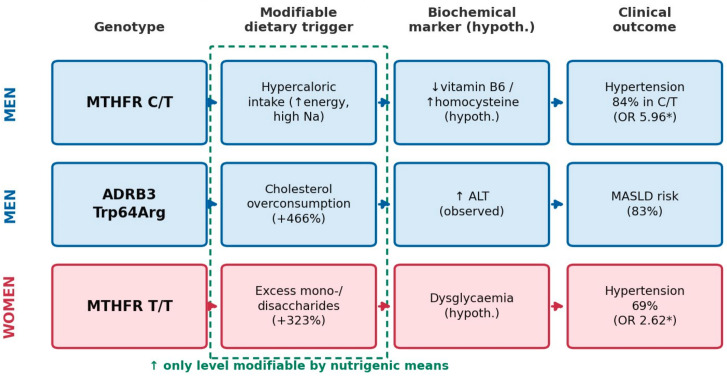
Hypothesised gene–diet–disease cascade in obesity (conceptual model): from genotype through a modifiable dietary trigger and a biochemical marker to the clinical outcome, shown separately for men (blue) and women (red). Only the dietary level is modifiable by nutrigenic means. Hcy, homocysteine; HTN, hypertension. This diagram represents a proposed conceptual model based on cross-sectional associations, not an experimentally validated causal pathway; temporal relationships were not established, and homocysteine and dysglycaemia pathways were not measured. * *p* < 0.05 (statistically significant).

Integrating the findings suggests a hypothesised pathogenic cascade linking genotype, a modifiable dietary trigger, a biochemical marker and a clinical outcome (Figure 9): MTHFR C/T in men → generalised hypercaloric intake (including a high absolute sodium load) → lowered/raised vitamin B6.

## 4. Discussion

To our knowledge, this study is among the first to suggest that the same polymorphism is associated with distinct dietary phenotypes—and distinct inheritance models—in men and women with obesity. Genotype thus modifies not only disease risk but actual food consumption, and it does so in a sex-specific manner. This reframes three well-studied polymorphisms as candidate sex-differentiated, dietarily actionable targets rather than universal risk markers, a hypothesis that remains to be tested prospectively.

### 4.1. MTHFR: Two Dietary Faces of the T-Allele

In men the T-allele produced a bimodal nutrient signature—hypercaloric in C/T and deficient in T/T—whereas in women T/T manifested as excessive mono-/disaccharide intake. The male dominant-heterozygous hypertension model (C/T, OR 5.96, especially below 40 years) is consistent with the role of the T-allele in hyperhomocysteinaemia and endothelial dysfunction [11,17] and with Turkish and meta-analytic evidence linking T/T with essential hypertension [17,18], with sex- and age-specific effects reported in a South African cohort [19]; the Saudi obesity–hypertension cohort reported comparable direction [20]. The female recessive model (T/T → hypertension) is directly supported by Wilson et al., who found higher central and peripheral blood pressure in female T/T carriers [10] and identified riboflavin (vitamin B2) status—a cofactor of MTHFR—as the critical modulator. A series of Irish randomised trials showed that targeted riboflavin (1.6 mg/day) lowers systolic blood pressure by 6–13 mmHg in T-allele carriers [21,22,23,24]. Notably, in our sample male T/T carriers had a riboflavin (B2) intake deficit (−26.4% vs. +14.4% in C/C) and lower serum vitamin B6 (8.04 vs. 11.26 µg/L; *p* = 0.004). This suggests a coherent mechanistic chain in men: the C/T-linked hypercaloric pattern delivers a high absolute sodium load and, in the deficient T/T state, an inadequate supply of the B-vitamin cofactors of one-carbon metabolism. Because riboflavin (as FAD) is the cofactor of MTHFR and vitamin B6 (as pyridoxal-5′-phosphate) is the cofactor of cystathionine-β-synthase in the transsulphuration pathway, a concurrent shortfall of both vitamins compounds the reduced enzymatic activity of the T-allele, elevating homocysteine and promoting endothelial dysfunction and blood-pressure elevation. The genotype is thereby converted from an immutable adverse marker into a modifiable target: correcting the dietary excess (energy and sodium) while repleting riboflavin, B6 and folate addresses both arms of the cascade—the haemodynamic load and the one-carbon deficit—by non-pharmacological means. The sex difference is biologically anchored in the vasoprotective and homocysteine-lowering actions of oestrogen via cystathionine-β-synthase, so that in women risk is realised only under the most severe genetic deficit (T/T) [25]. Non-confirmatory findings: Several a priori hypotheses were not confirmed, and reporting these negative results is essential for a balanced interpretation of the data. First, as plasma homocysteine was not measured in this study, the proposed one-carbon/homocysteine mechanism linking MTHFR, B-vitamins and blood pressure remains a hypothesis rather than a demonstrated pathway in this cohort. Second, the initially observed PPARG G/G–sodium association did not persist after harmonisation of sodium units and was therefore not retained as a finding. Third, no genuine additive polygenic effect on hypertension was demonstrable in either sex, and ADRB3 Trp64Arg carried no independent hypertension signal. Finally, many of the estimates derived from age- and subgroup-stratified analyses rest on small numbers and should therefore be considered exploratory.

### 4.2. PPARG G/G as a Hepatic (ALT) Marker in Young Women

PPARG G/G (Ala/Ala) in women is retained here as a marker of hepatic risk—elevated ALT in young carriers—rather than of dietary sodium. An earlier PPARG G/G–sodium association did not persist after harmonisation of sodium units across the two datasets (*p* = 0.47); the sodium signal in this cohort is instead carried by MTHFR in men, so sodium is reassigned as a male target and PPARG G/G is followed by hepatic (ALT) monitoring only. This extends the established nutrient-dependent regulation of PPARG phenotypes by dietary fat [14] and aligns with reports that PPARG associations with overweight and metabolic phenotypes are strongest—or exclusive—in women [7,12]. The parallel association of G/G with elevated ALT in young women points to a hepatic and blood-pressure (RAAS-linked) risk axis. Because sodium is directly modifiable, it remains a priority target; in this cohort, however, the genotype that identifies excess sodium intake is MTHFR C/T in men (3047 vs. 1609 mg Na/day; pairwise *p* = 0.003)—the same genotype that carries the male hypertension risk. This provides a more parsimonious dietary link between MTHFR C/T and blood pressure than the homocysteine pathway alone. Given the conflicting literature on the direction of Pro12Ala effects [26,27,28], this association requires confirmation in larger independent samples.

### 4.3. ADRB3: A Sex-Divergent “Mirror” of Intake and Metabolism

The ADRB3 Trp64Arg “mirror”—hyperphagia and cholesterol overconsumption in men and hypophagia in women—mirrors the biochemical divergence (higher ALT in men, lower uric acid in women). The male cholesterol signal (+466%) and the ALT elevation accord with Saudi dyslipidaemia data [29] and with Japanese evidence identifying rs4994 as a biomarker of steatotic liver disease in overweight/obese individuals [30], while meta-analysis confirms the Arg64–dyslipidaemia link [31]; conversely, protective associations with higher HDL-cholesterol [32] and associations with obesity in paediatric populations [33] have also been reported. A plausible mechanism is the reduced β3-adrenergic lipolytic activity of Arg64, prompting compensatory hyperphagia in men, whereas oestrogen-dependent modulation of appetite centres and hepatic PPARα/CYP4a-mediated protection in women may blunt or reverse the effect [34,35]. Russian data showing a higher minor-allele frequency in women [36] may further contribute to the observed sex asymmetry. The lower uric acid in female Arg64 carriers is a new observation requiring prospective confirmation.

### 4.4. Sex-Differentiated Nutritional Targets and Prevention Algorithm

**Figure 10:**
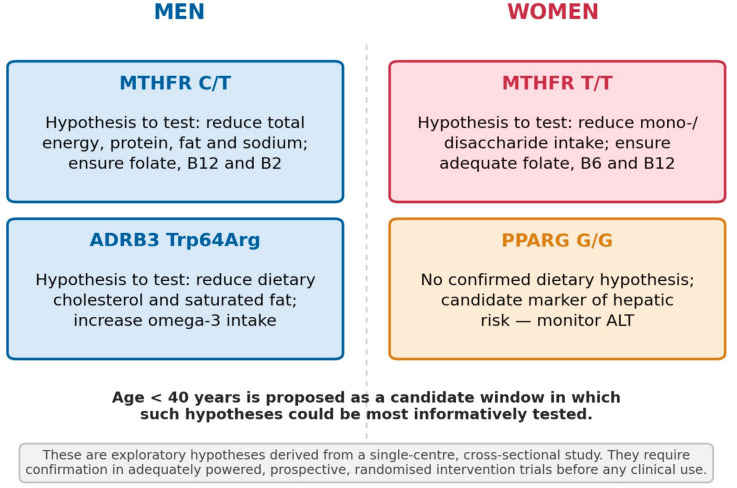
Proposed sex- and genotype-specific dietary hypotheses for future testing in obesity. These are candidate hypotheses derived from cross-sectional associations, not validated clinical targets, and require prospective interventional confirmation.

**Figure 11:**
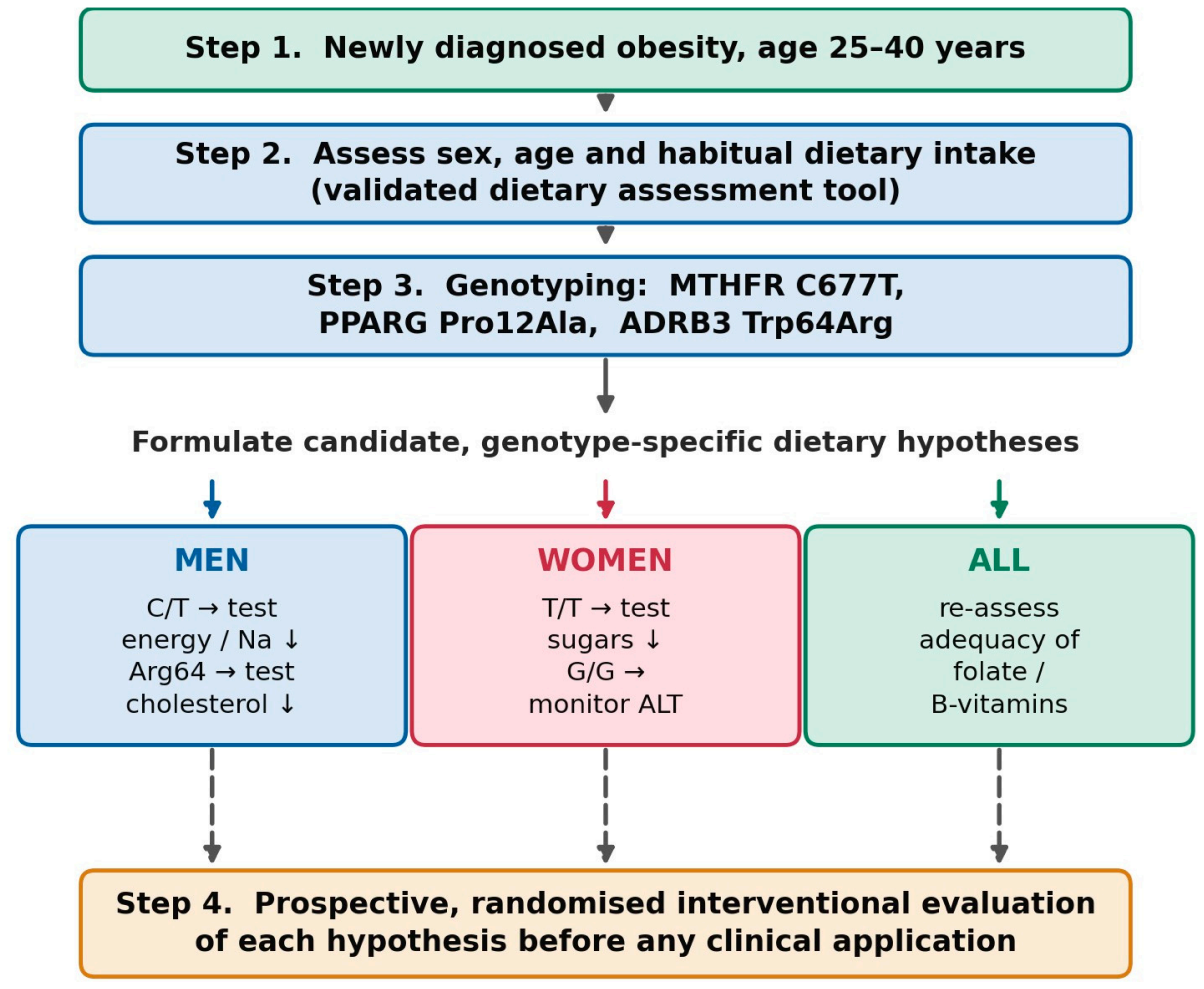
Conceptual framework for future genotype-informed dietary hypothesis testing in obesity, integrating sex, age and genotype. This scheme is a research proposal to structure future studies, not a validated clinical algorithm.

Two architectures of genetic risk emerged. In men, the cardiovascular signal rests on MTHFR C/T alone: combining it with ADRB3 showed no independent additional effect on hypertension (ADRB3 Arg64 carried no independent hypertension signal), so the two loci mark distinct dietary targets rather than an additive cardiovascular risk. In women, each gene likewise acts on its own target (MTHFR T/T → hypertension; PPARG G/G → ALT/hepatic risk). This sex × genotype specificity, rather than simple additive accumulation, is what a personalised scheme must capture [37]. Across all three polymorphisms, associations were maximal below 40 years, suggesting a priority window for future investigation before age-related risk saturation. On this basis we outline candidate sex- and genotype-specific dietary hypotheses (Figure 10) within a conceptual framework for future hypothesis testing (Figure 11). For men with C/T MTHFR the candidate hypotheses to be tested are reduced calories, protein, total fat and sodium with increased folate, B12 and B2; for men with Trp64Arg ADRB3, reduced cholesterol and SFA with increased omega-3. For women with T/T MTHFR the candidate hypothesis to be tested is reduced mono-/disaccharides with folate/B6/B12 repletion; women with PPARG G/G have no confirmed dietary target and are followed with hepatic (ALT) monitoring. Sodium restriction is therefore assigned to men carrying MTHFR C/T, not to women—an explicit sex-specific correction relative to earlier undifferentiated schemes.

### 4.5. Limitations

Several limitations should be acknowledged. The cross-sectional design limits the ability to establish temporal relationships between genotype, diet and outcomes; therefore, the proposed pathways should be interpreted as associations requiring prospective confirmation. The relatively small male subgroup (*n* = 83) and its genotype-stratified subsets limit statistical power; findings based on small subgroups are hypothesis-generating and require validation in larger cohorts. The single-centre design and ethnically homogeneous Russian cohort limit generalisability. Although occupational physical activity was uniformly low, leisure-time exercise was not quantified; future studies should include objective activity measurements. Dietary intake was assessed by self-report, which is subject to under-reporting in obesity. Menopausal status was not recorded, and residual confounding by unmeasured lifestyle factors (smoking, alcohol, socioeconomic status, education, medication use) cannot be excluded. With respect to the statistical framework, we applied Benjamini–Hochberg FDR correction within families of related comparisons, as described in Section 2.6. The two Bonferroni-surviving associations are treated as confirmatory. All other findings—including the female MTHFR T/T–mono/disaccharide association (*p* = 0.027, q = 0.189), the PPARG G/G–ALT association (*p* = 0.027, q = 0.054), the female MTHFR T/T–hypertension tendency (*p* = 0.035, q = 0.078), and the ADRB3 directional patterns in women (all q > 0.15)—did not reach the pre-specified FDR threshold of q < 0.05 and are therefore hypothesis-generating. These exploratory findings require independent validation in larger cohorts before any quantitative interpretation can be made. A particularly important caveat concerns the statistical stability of findings derived from small genotype subgroups. In men, the MTHFR T/T (*n* = 9) and ADRB3 Arg64 (*n* = 12) subgroups are small. Post hoc power calculations showed that in these subgroups, only very large effects (Cohen’s d > 1.0 for continuous outcomes) could be detected with confidence. Therefore, the absence of significant associations in these subgroups should not be interpreted as evidence of no effect, and the significant male ADRB3 Arg64–cholesterol excess should be viewed as provisional until replicated in larger cohorts. The same applies to the age-stratified analyses, where subgroup sizes were even smaller. Plasma homocysteine was not measured, so the proposed one-carbon pathway remains speculative. Finally, menopausal status was not recorded—a potential source of residual confounding in women around the menopause, when oestrogen-mediated protection against hyperhomocysteinaemia and hypertension diminishes—and medication use (antihypertensives, statins, vitamins) was not systematically recorded and could have influenced biochemical and clinical outcomes. Despite these limitations, the consistency of the sex-specific associations across multiple dietary and biochemical endpoints, together with their biological plausibility, supports the potential relevance of the identified hypotheses for future research. Prospective, multi-centre studies with pre-specified multiple-comparison correction and biomarker assessment are required to confirm these findings and to test the proposed hypotheses in interventional settings.

## 5. Conclusions

In patients with obesity, MTHFR rs1801133, PPARG rs1801282 and ADRB3 rs4994 were associated with sex-specific dietary phenotypes and inheritance models. MTHFR C/T was associated with a male hypercaloric pattern and a dominant-heterozygous hypertension model (OR 5.96), whereas T/T was associated with a female excessive mono-/disaccharide intake pattern and a recessive hypertension model (OR 2.62). ADRB3 Trp64Arg was associated with a dietary and biochemical “mirror”—cholesterol overconsumption and higher hepatic markers in men, and a blunted/inverse response in women. PPARG G/G was associated with elevated ALT in young women and is retained only as a marker of hepatic risk; the previously reported female sodium phenotype did not survive after unit harmonisation. In men, the cardiovascular risk was attributable to MTHFR C/T alone; combining MTHFR with ADRB3 in an unweighted allele-sum score revealed no genuine polygenic effect, as ADRB3 Trp64Arg carried no independent hypertension signal. In women, each gene was associated with its own target. Associations were most pronounced below 40 years, supporting the identification of this age period as a candidate priority window for gene-guided dietary prevention. Collectively, these associations give rise to specific, testable hypotheses—namely, that dietary cholesterol restriction in male carriers of the ADRB3 Arg64 allele, restriction of mono- and disaccharides in female MTHFR T/T homozygotes, and energy and sodium restriction in male MTHFR C/T heterozygotes may confer a reduction in metabolic risk. It must be emphasised, however, that these are working hypotheses rather than clinical recommendations. Their translation into clinical practice requires confirmation in adequately powered, prospective, randomised controlled intervention trials with predefined dietary protocols and hard metabolic endpoints. As this was a cross-sectional study, these associations do not establish causality, and prospective interventional validation is warranted.

## Figures and Tables

**Table 1:** General characteristics of patients with obesity with complete anthropometric and biochemical data (*n* = 348; Me [Q1; Q3]).

Parameter	Men (*n* = 83)	Women (*n* = 265)	*p*
Age, years	37.0 [26.5; 42.0]	37.0 [28.0; 47.0]	0.377
BMI, kg/m^2^	42.5 [36.4; 47.8]	37.3 [33.3; 44.7]	0.006
Fat mass, %	41.0 [36.3; 45.5]	49.3 [42.8; 51.7]	<0.001
Glucose, mmol/L	5.14 [4.79; 5.75]	5.16 [4.75; 5.63]	0.705
Total cholesterol, mmol/L	4.95 [3.96; 5.83]	4.80 [4.16; 5.76]	0.996
LDL-C, mmol/L	3.58 [2.71; 4.11]	3.37 [2.88; 4.09]	0.530
HDL-C, mmol/L	1.00 [0.90; 1.10]	1.20 [1.00; 1.40]	<0.001
Triglycerides, mmol/L	1.43 [0.96; 2.09]	1.11 [0.83; 1.60]	0.015
Uric acid, µmol/L	474 [378; 531]	335 [285; 389]	<0.001
ALT, U/L	40.0 [26.9; 55.5]	18.0 [14.0; 26.3]	<0.001
AST, U/L	25.0 [21.0; 46.1]	18.0 [14.3; 23.1]	<0.001
Basal metabolic rate, kcal/day	2132 [1861; 2680]	1738 [1477; 2029]	<0.001

Quantitative variables were compared with the Mann–Whitney U test, categorical variables with the chi-square test. ALT, alanine aminotransferase; AST, aspartate aminotransferase; BMI, body mass index; HDL-C/LDL-C, high-/low-density lipoprotein cholesterol.

**Table 2:** Habitual nutrient intake in men with obesity by MTHFR rs1801133 genotype; median deviation from recommended values (%, *n* = 83).

Nutrient	C/C (*n* = 44)	C/T (*n* = 30)	T/T (*n* = 9)	*p* (KW)	*p* (rec.)
Energy	+48.5	+83.8	+19.3	0.074	0.211
Protein	+45.2	+71.1	−6.6	0.042	0.043 *
Fat	+79.4	+111.9	+18.6	0.072	0.063
Carbohydrate	−29.4	−25.3	−46.4	0.167	0.099
Dietary fibre	−51.6	−168.9	−55.3	0.053	0.129
Sodium	+63.7	+134.3	+23.8	0.007 **	0.021 (C/T vs. T/T 0.003 **)
Magnesium	+5.7	+55.8	+10.8	0.170	0.683
Iron	+88.7	+155.6	+64.9	0.076	0.208
Phosphorus	+88.8	+127.7	+39.7	0.033	0.048 (C/T vs. T/T 0.012 *)
Potassium	+49.0	+67.6	+18.7	0.170	0.125
Vitamin B2	−12.8	+18.6	−24.1	0.025	0.077

KW, Kruskal–Wallis test; “rec.”, recessive model (T/T vs. C/C + C/T), Mann–Whitney U test. * pairwise Bonferroni-significant for three groups (*p* < 0.017), ** *p* < 0.017 (Bonferroni-corrected for the pairwise comparison C/T vs. T/T).

**Table 3:** Absolute habitual nutrient intakes in men with obesity by MTHFR rs1801133 genotype: medians and interquartile ranges.

Nutrient	C/C—Me [Q1; Q3]	C/T—Me [Q1; Q3]	T/T—Me [Q1; Q3]
Energy, kcal	3195 [2203; 3872]	3904 [2779; 4439]	2505 [2149; 2928]
Protein, g	128 [98.3; 151]	148 [114; 183]	85.0 [76.2; 116]
Fat, g	156 [108; 180]	186 [137; 230]	133 [119; 139]
Carbohydrate, g	243 [195; 396]	279 [194; 398]	226 [194; 288]
Mono-/disaccharides, g	108 [51.1; 204]	108 [70.2; 229]	124 [92.5; 162]
Fibre, g	11.8 [9.66; 16.5]	18.5 [12.2; 21.3]	9.11 [5.97; 10.1]
Cholesterol, mg	419 [286; 853]	563 [381; 973]	304 [196; 709]
SFA, g	55.2 [28.7; 64.1]	58.0 [39.8; 76.9]	45.4 [35.4; 51.2]
Omega-3, g	2.29 [0.75; 4.21]	2.80 [0.99; 5.74]	2.92 [2.04; 3.85]
Omega-6, g	16.3 [8.61; 37.2]	21.2 [10.7; 41.8]	23.4 [18.6; 28.0]
Sodium, mg Na	2128 [1644; 3375]	3045 [2368; 3836]	1609 [1408; 1932]
Potassium, mg	3726 [2901; 4685]	4191 [3198; 5911]	2969 [2797; 3430]
Calcium, mg	876 [643; 1474]	1187 [880; 1781]	899 [839; 1234]
Magnesium, mg	336 [264; 493]	485 [353; 734]	310 [264; 375]
Iron, mg	18.8 [13.6; 25.7]	25.6 [18.1; 36.4]	14.8 [13.3; 19.7]
Phosphorus, mg	1612 [1109; 2408]	2061 [1512; 2615]	1323 [1261; 1788]
Vitamin A, µg	878 [662; 1697]	1321 [831; 1994]	988 [804; 1217]
Vitamin B1, mg	1.14 [0.79; 1.76]	1.56 [1.10; 2.14]	0.97 [0.87; 1.39]
Vitamin B2, mg	1.57 [1.28; 2.55]	2.13 [1.80; 3.70]	1.33 [1.23; 2.03]
Vitamin B3, mg	19.1 [11.5; 26.5]	29.5 [19.6; 38.2]	15.7 [14.4; 26.8]
Vitamin C, mg	125 [83.1; 192]	172 [125; 307]	156 [96.2; 188]

**Table 4:** Habitual nutrient intake in women with obesity by MTHFR rs1801133 genotype; median deviation from recommended values (%, *n* = 265).

Nutrient	C/C (*n* = 131)	C/T (*n* = 98)	T/T (*n* = 36)	*p* (KW)	*p* (rec.)
Mono-/disaccharides	+195.6	+146.2	+323.4	0.079	0.027
Dietary fibre	−55.3	−40.6	−44.9	0.095	0.768
Potassium	+26.3	+32.7	+48.1	0.302	0.143
Omega-3 PUFA	+81.7	+31.7	+97.1	0.110	0.077
Vitamin A	+26.4	+20.9	+41.8	0.169	0.103
Vitamin B1	−37.0	−38.7	−28.0	0.266	0.109

KW, Kruskal–Wallis test; “rec.”, recessive model (T/T vs. C/C + C/T). The mono-/disaccharide association in the recessive model (*p* = 0.027) yielded FDR q = 0.189 across the 12 tests in this family and is therefore exploratory (see Section 2.6 for details).

**Table 5:** Absolute habitual nutrient intakes in women with obesity by MTHFR rs1801133 genotype: medians and interquartile ranges.

Nutrient	C/C—Me [Q1; Q3]	C/T—Me [Q1; Q3]	T/T—Me [Q1; Q3]
Energy, kcal	2206 [1798; 3285]	2225 [1834; 3329]	2378 [1908; 2921]
Protein, g	96.2 [68.1; 121]	98.4 [69.9; 143]	93.2 [69.0; 135]
Fat, g	112 [87.2; 154]	112 [85.0; 170]	113 [91.1; 160]
Carbohydrate, g	185 [132; 270]	203 [144; 268]	203 [153; 304]
Mono-/disaccharides, g	82.3 [52.6; 132]	84.7 [53.6; 156]	107 [81.3; 188]
Fibre, g	9.33 [6.38; 16.1]	12.0 [8.30; 18.0]	11.3 [7.16; 16.4]
Cholesterol, mg	380 [216; 605]	437 [233; 611]	315 [207; 466]
SFA, g	36.8 [25.8; 48.9]	34.4 [27.4; 57.9]	38.9 [29.2; 55.7]
Omega-3, g	2.20 [0.72; 3.85]	1.81 [0.74; 3.46]	2.74 [1.67; 4.08]
Omega-6, g	18.6 [7.15; 33.2]	14.6 [5.43; 28.8]	19.8 [9.99; 29.0]
Sodium, mg Na	1672 [1188; 2337]	1715 [1164; 2514]	1625 [1074; 2640]
Potassium, mg	3158 [2477; 4080]	3317 [2303; 4798]	3702 [2662; 5164]
Calcium, mg	911 [636; 1225]	897 [548; 1367]	974 [674; 1562]
Magnesium, mg	328 [249; 473]	342 [226; 501]	349 [273; 492]
Iron, mg	16.0 [11.9; 23.5]	16.3 [12.1; 22.5]	16.8 [13.3; 24.0]
Phosphorus, mg	1382 [1013; 1830]	1267 [966; 2072]	1406 [1083; 1961]
Vitamin A, µg	1057 [785; 1640]	1039 [540; 1605]	1276 [725; 1925]
Vitamin B1, mg	0.94 [0.65; 1.36]	0.91 [0.66; 1.36]	1.08 [0.88; 1.42]
Vitamin B2, mg	1.60 [1.17; 2.15]	1.56 [1.12; 2.23]	1.69 [1.19; 2.33]
Vitamin B3, mg	16.9 [11.0; 23.9]	17.6 [11.7; 26.0]	16.7 [10.1; 24.5]
Vitamin C, mg	125 [87.5; 201]	148 [80.5; 250]	165 [120; 281]

**Table 6:** Absolute habitual nutrient intakes by PPARG rs1801282 (Pro12Ala) genotype in women with obesity.

Nutrient	C/C (*n* = 167) Me [Q1; Q3]	C/G (*n* = 80) Me [Q1; Q3]	G/G (*n* = 18) Me [Q1; Q3]	*p* (KW)
Energy, kcal	2206 [1829; 3276]	2229 [1837; 3070]	2286 [1891; 3399]	0.902
Protein, g	96.4 [69.0; 132]	94.9 [69.1; 121]	110 [81.8; 123]	0.509
Fat, g	110 [85.0; 166]	113 [89.5; 153]	124 [106; 169]	0.285
Carbohydrate, g	191 [138; 282]	194 [144; 259]	197 [134; 281]	0.796
Mono-/disaccharides, g	92.3 [53.2; 138]	89.2 [65.4; 171]	81.4 [50.3; 134]	0.359
Fibre, g	10.5 [7.02; 16.4]	10.6 [7.05; 16.3]	12.4 [7.00; 27.6]	0.524
Cholesterol, mg	354 [208; 615]	400 [255; 513]	325 [196; 594]	0.534
SFA, g	35.4 [27.3; 52.8]	35.6 [26.6; 49.1]	36.9 [30.2; 45.0]	0.813
Omega-3, g	2.12 [0.76; 3.73]	2.23 [1.18; 3.27]	2.59 [0.98; 5.05]	0.735
Omega-6, g	17.1 [6.30; 29.9]	18.6 [7.88; 29.0]	19.8 [6.77; 44.7]	0.675
Sodium, mg Na	1644 [1161; 2412]	1719 [1117; 2577]	1877 [1609; 2683]	0.413
Potassium, mg	3252 [2464; 4697]	3217 [2493; 3965]	3004 [2170; 5394]	0.945
Calcium, mg	900 [597; 1291]	915 [716; 1154]	878 [479; 1355]	0.743
Magnesium, mg	341 [246; 502]	323 [261; 473]	269 [212; 489]	0.712
Iron, mg	16.4 [12.7; 22.6]	16.5 [12.7; 22.4]	14.7 [10.3; 26.4]	0.676
Phosphorus, mg	1346 [976; 2044]	1400 [1108; 1753]	1218 [878; 1848]	0.711
Vitamin A, µg	1080 [712; 1640]	1088 [738; 1683]	944 [540; 1781]	0.678
Vitamin B1, mg	0.94 [0.66; 1.36]	0.99 [0.66; 1.39]	1.15 [0.76; 1.34]	0.706
Vitamin B2, mg	1.58 [1.15; 2.19]	1.65 [1.24; 2.12]	1.39 [0.93; 2.24]	0.624
Vitamin B3, mg	17.4 [11.0; 25.0]	17.1 [10.3; 25.5]	17.2 [12.7; 22.6]	0.908
Vitamin C, mg	137 [86.1; 242]	145 [87.5; 225]	127 [110; 225]	0.914

Note: values are median [Q1; Q3] of absolute daily intake. p (KW)—Kruskal–Wallis test across the three genotypes. Sodium is reported as elemental sodium (mg/day); 1 g NaCl = 393.4 mg Na. No nutrient differed significantly between PPARG genotypes in women (all *p* > 0.05), consistent with the absence of a PPARG-associated dietary phenotype.

**Table 7:** Absolute habitual nutrient intakes by PPARG rs1801282 (Pro12Ala) genotype in men with obesity.

Nutrient	C/C (*n* = 53) Me [Q1; Q3]	C/G (*n* = 25) Me [Q1; Q3]	G/G (*n* = 5) Me [Q1; Q3]	*p* (KW)
Energy, kcal	3217 [2322; 4439]	3198 [2415; 4132]	3759 [3672; 3846]	0.937
Protein, g	125 [95.6; 158]	136 [99.0; 165]	164 [147; 180]	0.523
Fat, g	150 [118; 205]	164 [134; 199]	200 [198; 202]	0.163
Carbohydrate, g	245 [190; 385]	242 [197; 396]	322 [320; 323]	0.457
Mono-/disaccharides, g	112 [72.3; 231]	113 [54.2; 190]	41.0 [30.6; 51.5]	0.127
Fibre, g	11.8 [9.06; 17.6]	15.0 [9.95; 21.0]	17.9 [16.1; 19.7]	0.484
Cholesterol, mg	470 [272; 806]	429 [330; 1036]	463 [425; 501]	0.358
SFA, g	51.2 [35.2; 73.4]	54.2 [35.3; 70.7]	64.5 [63.7; 65.3]	0.764
Omega-3, g	2.80 [0.81; 5.51]	2.29 [0.94; 4.71]	2.06 [1.85; 2.28]	0.531
Omega-6, g	22.1 [8.30; 37.9]	17.7 [10.3; 37.7]	16.5 [16.2; 16.8]	0.839
Sodium, mg Na	2415 [1644; 3348]	2553 [1814; 3635]	2924 [1903; 3982]	0.101
Potassium, mg	3963 [2938; 5020]	3713 [3123; 5737]	3059 [3001; 3117]	0.536
Calcium, mg	965 [677; 1418]	917 [638; 1716]	1079 [1028; 1129]	0.908
Magnesium, mg	439 [281; 543]	405 [253; 592]	302 [299; 305]	0.386
Iron, mg	20.5 [13.8; 25.4]	25.5 [14.9; 32.3]	21.7 [20.1; 23.3]	0.587
Phosphorus, mg	1686 [1310; 2350]	1692 [1197; 2428]	1382 [1339; 1426]	0.985
Vitamin A, µg	1007 [777; 1732]	1217 [724; 1902]	434 [425; 443]	0.103
Vitamin B1, mg	1.19 [0.83; 2.06]	1.39 [1.01; 1.76]	1.64 [1.48; 1.80]	0.795
Vitamin B2, mg	2.01 [1.38; 2.62]	1.92 [1.26; 3.08]	1.48 [1.43; 1.52]	0.690
Vitamin B3, mg	23.7 [14.6; 32.5]	19.9 [12.1; 30.8]	22.9 [19.6; 26.2]	0.636
Vitamin C, mg	155 [89.8; 232]	128 [89.8; 324]	113 [94.6; 131]	0.673

Note: values are median [Q1; Q3] of absolute daily intake. p (KW)—Kruskal–Wallis test. Sodium as elemental sodium (mg/day); 1 g NaCl = 393.4 mg Na. The G/G subgroup in men was very small (*n* = 5); the corresponding medians are shown for completeness but are not interpretable. No robust nutrient differences were observed between PPARG genotypes in men.

**Table 8:** Genotype–nutrient associations under three regression models, estimated by linear regression of log-transformed intakes on genotype: unadjusted, adjusted for age and BMI, and adjusted for log total energy intake.

Association (Sex)	Nutrient	Crude % (*p*)	+Age, BMI % (*p*)	+Energy % (*p*)
MTHFR C/T (men)	Sodium	+36% (0.020)	+27% (0.162)	+16% (0.189)
	Protein	+27% (0.034)	+29% (0.119)	+9% (0.208)
	Fat	+27% (0.031)	+31% (0.103)	−1% (0.892)
	Phosphorus	+24% (0.047)	+24% (0.164)	+6% (0.286)
ADRB3 Arg64 (men)	Cholesterol	+98% (0.009)	+81% (0.026)	+47% (0.035)
MTHFR T/T (women)	Mono-/disacch.	+28% (0.054)	+9% (0.559)	+20% (0.072)

**Table 9:** Difference in habitual nutrient intake between Arg64 carriers and Trp/Trp for ADRB3 rs4994, by sex (Δ of medians, %).

Nutrient	Men Δ (Arg − Trp), %	Men *p*	Women Δ (Arg − Trp), %	Women *p*
Cholesterol	+313 **	0.012	+8	0.803
Omega-6 PUFA	+265	0.091	−51	0.169
SFA	+155	0.259	−38	0.201
Omega-3 PUFA	+173	0.152	−65	0.134
Iron	+157	0.055	−7	0.672
Potassium	+78	0.160	−16	0.983
Phosphorus	+74	0.292	+2	0.635
Fat	+41	0.362	−24	0.249
Carbohydrate	+31	0.055	−7	0.406

Men: Trp/Trp *n* = 61, Trp64Arg *n* = 22. Women: Trp/Trp *n* = 209, Trp64Arg *n* = 56. ** *p* < 0.017; (Mann–Whitney U test). None of the associations in women reached FDR q < 0.05 (all q > 0.15); all female ADRB3 findings are therefore exploratory (see Section 2.6 for details).

**Table 10:** Absolute habitual nutrient intakes by ADRB3 rs4994 (Trp64Arg) genotype and sex: medians and interquartile ranges.

Nutrient	Men Trp/Trp	Men Trp64Arg	Women Trp/Trp	Women Trp64Arg
Energy, kcal	3688 [2619; 4843]	4760 [3406; 5719]	2674 [1866; 3603]	2378 [1504; 3444]
Protein, g	126 [85.0; 161]	181 [111; 207]	89.9 [62.7; 133]	96.4 [60.8; 126]
Fat, g	155 [126; 220]	201 [139; 267]	125 [88.8; 195]	105 [79.1; 172]
Carbohydrate, g	298 [198; 437]	427 [381; 470]	209 [145; 302]	180 [121; 284]
Mono-/disaccharides, g	190 [99.9; 244]	197 [164; 230]	117 [78.1; 178]	97.0 [72.3; 178]
Fibre, g	10.1 [7.81; 14.9]	15.6 [8.93; 27.7]	8.62 [6.32; 14.3]	9.95 [6.44; 14.6]
Cholesterol, mg	380 [201; 607]	849 [429; 1005]	327 [207; 516]	339 [175; 469]
SFA, g	56.7 [35.4; 75.4]	80.1 [49.2; 103]	38.8 [27.4; 62.7]	36.7 [23.1; 49.9]
Omega-3, g	3.94 [2.41; 5.68]	6.01 [4.02; 10.2]	3.12 [2.17; 5.13]	2.34 [1.93; 4.23]
Omega-6, g	27.1 [18.6; 46.0]	51.0 [35.6; 59.0]	25.6 [18.4; 43.2]	21.0 [15.7; 36.9]
Sodium, mg Na	2018 [1542; 2836]	3139 [2211; 6216]	1566 [1046; 2412]	1471 [1054; 2179]
Potassium, mg	4106 [3186; 6008]	6063 [3725; 8082]	3668 [2580; 5376]	3274 [2740; 5334]
Calcium, mg	1194 [769; 1748]	1717 [868; 2004]	1027 [781; 1589]	960 [801; 1343]
Magnesium, mg	484 [324; 670]	713 [471; 908]	368 [269; 542]	324 [277; 495]
Iron, mg	23.3 [16.4; 35.2]	37.4 [26.4; 47.1]	18.2 [13.4; 26.3]	17.0 [13.9; 27.1]
Phosphorus, mg	2061 [1363; 2816]	2780 [1593; 3289]	1564 [1093; 2221]	1581 [1014; 1954]
Vitamin A, µg	1507 [924; 1940]	1926 [797; 3481]	1385 [869; 1835]	1378 [944; 1927]
Vitamin B1, mg	1.41 [0.97; 2.25]	2.43 [1.62; 2.73]	1.10 [0.78; 1.50]	1.04 [0.71; 1.42]
Vitamin B2, mg	1.96 [1.33; 2.70]	3.10 [1.36; 3.27]	1.71 [1.15; 2.37]	1.53 [1.14; 2.11]
Vitamin B3, mg	22.3 [15.9; 32.4]	29.1 [25.6; 50.3]	17.2 [10.9; 25.3]	15.5 [9.96; 24.9]
Vitamin C, mg	157 [121; 283]	280 [133; 491]	166 [110; 254]	223 [102; 302]

**Table 11:** Multivariable logistic-regression models for the genotype–hypertension associations, showing crude and covariate-adjusted odds ratios with exact event counts. Models: crude (unadjusted); adjusted for age and BMI; additionally adjusted for total energy intake.

Association	Model	*n* (Events)	OR [95% CI]	*p*
MTHFR C/T → hypertension (men)	Crude	55 (33)	5.96 [1.48; 24.08]	0.010
	+age, BMI	55 (33)	6.14 [1.48; 25.53]	0.013
	+age, BMI, energy	43 (28)	3.31 [0.69; 15.86]	0.134
MTHFR T/T → hypertension (women)	Crude (Fisher)	26 (18)	2.62 [1.07; 6.43]	0.035
	+age, BMI	167 (83)	2.62 [0.97; 7.09]	0.058
	+age, BMI, energy	137 (69)	2.27 [0.80; 6.42]	0.124

## Data Availability

The data presented in this study are available upon reasonable request from the corresponding author. The data are not publicly available due to privacy and ethical restrictions.

## Abbreviations

ADRB3	β3-Adrenergic Receptor Gene
ALT/AST	Alanine/Aspartate Aminotransferase
BMI	Body Mass Index
HDL-C/LDL-C	High-/Low-Density Lipoprotein Cholesterol
MASLD	Metabolic Dysfunction-Associated Steatotic Liver Disease
MTHFR	Methylenetetrahydrofolate Reductase Gene
OR	Odds Ratio
PPARG	Peroxisome Proliferator-Activated Receptor Gamma Gene
PUFA/SFA	Poly-unsaturated/Saturated Fatty Acids
RAAS	Renin–Angiotensin–Aldosterone System
